# A Sequence of Developmental Events Occurs Underneath Growing *Bacillus subtilis* Pellicles

**DOI:** 10.3389/fmicb.2019.00842

**Published:** 2019-04-26

**Authors:** Lisa M. Lee, Gili Rosenberg, Shmuel M. Rubinstein

**Affiliations:** ^1^John A. Paulson School of Engineering and Applied Sciences, Harvard University, Cambridge, MA, United States; ^2^Kavli Institute for Bionano Science and Technology, Cambridge, MA, United States; ^3^Department of Molecular Genetics, Weizmann Institute of Science, Rehovot, Israel

**Keywords:** pellicles, biofilms, development, collective behavior, flagellar motility

## Abstract

Biofilms are structured communities of bacteria that exhibit complex spatio-temporal dynamics. In liquid media, *Bacillus subtilis* produces an opaque floating biofilm, or a pellicle. Biofilms are generally associated with an interface, but the ability of *Bacillus subtilis* to swim means the bacteria are additionally able to reside within the liquid phase. However, due to imaging complications associated with the opacity of pellicles, the extent to which bacteria coexist within the liquid bulk as well as their behavior in the liquid is not well studied. We therefore develop a high-throughput imaging system to image underneath developing pellicles. Here we report a well-defined sequence of developmental events that occurs underneath a growing pellicle. Comparison with bacteria deficient in swimming and chemotaxis suggest that these properties enable collective bacterial swimming within the liquid phase which facilitate faster surface colonization. Furthermore, comparison to bacteria deficient in exopolymeric substances (EPS) suggest that the lack of a surface pellicle prevents further developmental steps from occurring within the liquid phase. Our results reveal a sequence of developmental events during pellicle growth, encompassing adhesion, conversion, growth, maturity, and detachment on the interface, which are synchronized with the bacteria in the liquid bulk increasing in density until the formation of a mature surface pellicle, after which the density of bacteria in the liquid drops.

## 1. Introduction

Bacteria in nature are mostly found in the form of biofilms, or structurally complex communities encased in an extracellular matrix (Sutherland, 2001; Branda et al., 2005; Kolter and Greenberg, 2006; Aguilar et al., 2007; Flemming and Wingender, 2010) at a surface (O'Toole et al., 2000; Ghannoum, 2004). The formation of a biofilm involves intricate spatio-temporal heterogeneity, displaying features such as spatial patterns of gene expression (Rubinstein et al., 2012; Srinivasan et al., 2018), cellular differentiation (Stoodley et al., 2002), and morphology (McLoon et al., 2011) in a manner analogous to multicellular organisms (Shapiro, 1998).

The paradigm for biofilm formation involves planktonic bacteria attaching to a surface, inducing cell-cell adhesion, proliferating, maturing, and then finally dispersing back into planktonic bacteria, which may then repeat the cycle (Stoodley et al., 2002). *Bacillus subtilis* is a convenient model organism for studying the mechanisms of biofilm formation. Although biofilms can form at nearly any interface, most *B. subtilis* biofilm studies to date focus on the growth of a biofilm on agar gels, an air-solid interface, where the biofilm grows, thickens, and spreads radially outwards from its inoculation zone as a consequence of osmotic pressure gradients in the underlying agar (Seminara et al., 2012) and mechanical pushing resulting from the growth and division of the bacteria (Dervaux et al., 2014). Additionally, waves of gene expression propagate across immobilized cells in a self-similar manner behind the growing front (Srinivasan et al., 2018). However, *B. subtilis* also forms robust biofilms at an air-liquid interface, called pellicles. Despite many similarities between biofilms grown at an air-solid interface and pellicles grown at an air-liquid interface, there are marked differences as well, one being that pellicles initially grow as a thin layer over the entire interface before thickening and wrinkling (Kobayashi, 2007), which completes their entire lifecycle. Although biofilms grown on agar also start as a thin layer of cells that thicken in their inoculation zone, the majority of their growth is directed radially outwards from the inoculation area. Additionally, biofilms grown on agar are restricted to the interface since the bacteria are unable to penetrate the underlying substrate, but the ability of *B. subtilis* to swim means the bacteria within pellicles interact with the underlying liquid medium. In dense suspensions of bacteria in liquid, collective bacterial swimming phenomena such a bioconvection (Jánosi et al., 1998) and whirls and jets (Mendelson et al., 1999) have been reported, giving rise to characteristic length and time scales due to competing environmental cues and bacterial swimming interactions. As such, it is reasonable to hypothesize that the growth of a *B. subtilis* floating pellicle involves not only the bacteria at the interface, but also several stages of bacterial collective activity below the pellicle in the liquid bulk (Ardré et al., 2015; Hölscher et al., 2015; Steinberg et al., 2018).

On the cellular level, the developmental checkpoints of a growing pellicle have been extensively described by Kobayashi (2007). The morphological changes of bacteria in the liquid during pellicle formation start off as rapidly swimming individual cells, forming cell chains after 6 h that then increase in number to become woven string-like structures at 8 h, and then finally separate from woven structures back into cells again. This entire cycle describes the first 10 h, all before the pellicle has fully formed on the surface. The final stage also corresponds to a hazy appearance at the bottom of the well (Kobayashi, 2007), later reported to correspond to a vortex-like collective motility by aggregates of motile cells and EPS producers (Steinberg et al., 2018). Since the development of pellicles involves both the liquid phase as well as the interface, the pellicle developmental picture may be more complicated than the standard scheme for biofilm formation, which involves only the interface.

The dynamics and physiology of pellicle formation is complex. Capturing these processes requires direct visualization and continuous measurements of many samples over several days, which is challenging and costly with commercially available microscopes. In this study, we develop a high throughput imaging setup to simultaneously image, in high resolution, the liquid underneath 12 developing pellicles in any given experiment. We identify 7 distinct stages of pellicle formation which encompass bacteria reproducing to a high density in the liquid, rising through the liquid, colonizing the surface, becoming suppressed within the liquid, and finally biofilm dispersal. We then compare these steps to the developmental stages of several mutants to find that chemotaxis and motility contribute to faster colonization of the surface via dynamic collective streaks in the liquid. Additionally, we find that the lack of a stable surface pellicle in an EPS mutant is associated with the bacteria density in the liquid continuing to increase, in contrast to the drop in bacteria density that occurs in the liquid under robust surface pellicles.

## 2. Materials and Methods

**Figure 1:**
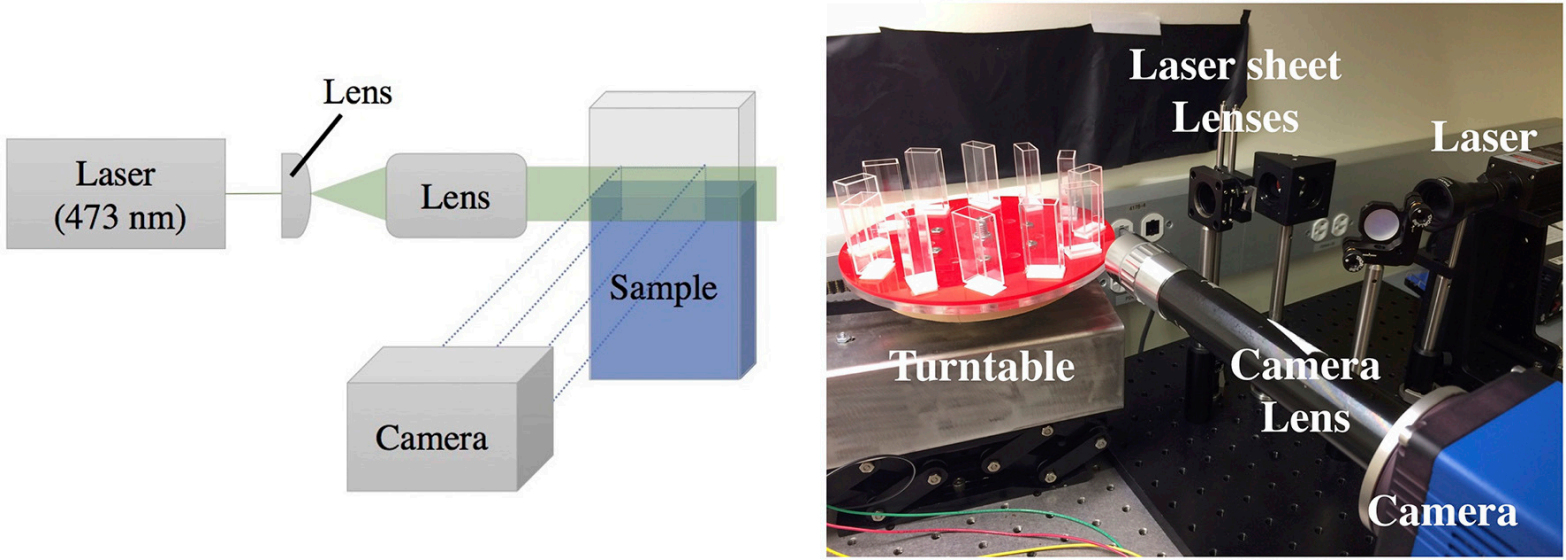
Laser sheet setup to image the activity occurring underneath a pellicle growing at an air-liquid interface. **Left**: Laser sheet diagram, where a laser is expanded through a system of lenses to illuminate a vertical cross section of the sample, visualized with a SCMOS imaging sensor using custom optics. **Right**: A photograph of the setup. The imaging setup is coupled to a turntable that allows high-throughput time-lapse imaging of up to 12 samples simultaneously.

The opacity of pellicles makes observing activity within the liquid below them a challenge; therefore, they are less studied than other biofilm phenotypes. In order to overcome the imaging limitations associated with standard top-down or bottom-up microscopy or imaging through the curved side of a well plate, we grow our pellicles in rectangular glass cuvettes (45 × 12.5 × 22.5 mm) covered with parafilm and image them from the side. To achieve finer detail associated with a thin cross-sectional layer under the pellicle, a laser (473 nm) sheet system illuminates a vertical cross section underneath the pellicle. Some experiments are conducted with the laser sheet, illuminating an area ~5 mm across and ~3 mm below the surface and ~1 mm thick. Other experiments image the full cuvette, either illuminated from the back with a collimated LED light source, or illuminated from the front with an LED light source, with black paper attached to the back of the cuvette. The setup is synced to a camera and built atop a turntable that can run twelve experiments simultaneously in identical conditions as shown in Figure 1, allowing for sufficient multiplexing in the recording of growing pellicles. We also perform optical density (OD) measurements by projecting a collimated LED light source behind the cuvette, with pixel intensities calibrated using solutions that were first measured with a commercial OD meter (see Supplementary Material). We therefore also report the spatially resolved average bacterial densities within the liquid as the pellicle develops.

*B. subtilis* NCIB 3610 cells were grown in LB from a single colony isolated from LB plates to a mid-logarithmic phase (5h at 37°C with shaking) and diluted to OD 0.6. The pellicle assays were inoculated in standard MSGg medium (Branda et al., 2001) in a 1:1,000 ratio, with 3 mL of MSGg per cuvette. Pellicles in culture were grown at room temperature (23°C), and photos of the pellicles were acquired using a PCO edge 4.2 SCMOS camera with a Canon Macro lens for full well images, and a Mitutoyo M Plan Apo 2x lens for images illuminated with a laser sheet. Each cuvette was imaged every 10 min.

Fluorescence measurements were conducted on a modified NCIB 3610 *B. subtilis* strain (MTC871) (Wang et al., 2016; Srinivasan et al., 2018) harboring three transcriptional fusions of distinct fluorescent proteins with promoters of (i) motile genes expressing the *hag* gene (Mirel and Chamberlin, 1989), (ii) EPS matrix producing cells that express the *tapA-sipW-tasA* operon (Romero et al., 2014), and (iii) sporulating cells that express the *sspB* gene (Mason et al., 1988); we hereafter refer to this strain as the triple-reporter. Fluorescence measurements were performed using a Zeiss Axiozoom V16 microscope and PlanNeoFluar 1.0x lens visualizing from the side of the cuvette using a mirror.

## 3. Results

### 3.1. A Defined Sequence of Developmental Events Occurring Under the Developing Pellicle

**Figure 2:**
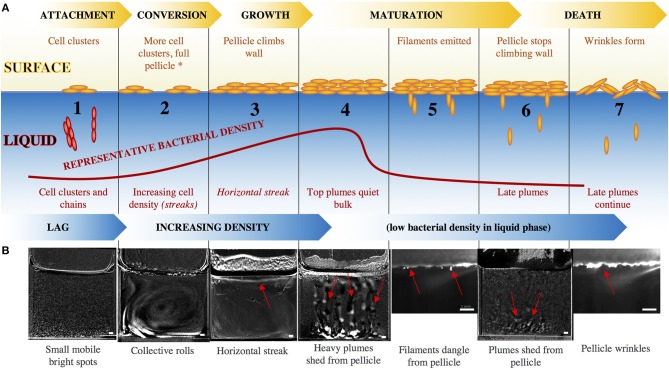
**(A)** Surface pellicle formation progresses through the standard steps of attachment, conversion, growth, maturation, and dispersal, while the bacterial density in the liquid bulk increases until the formation of a mature surface pellicle, after which the density of bacteria in the liquid drops. The red curve indicates representative OD of the bacteria in the liquid, and captioned are the steps. Beyond stage 2, the motility/chemotaxis mutants maintain the same surface events as the wild-type, but have altered liquid bulk events, indicated by the italics. The asterisk at stage 2 signals the point beyond which EPS mutants do not progress. **(B)** Snapshots of the series of events in the development of a wild-type pellicle. Smaller images (stages 5, 7) are a zoomed-in view using the laser sheet, and larger images (stages 1–4, 6) are views of the entire well with the previous time frame subtracted from the current time frame to emphasize features. Red arrows point to the relevant features. Scale bar is 1 mm. For larger images, see Figure S5.

At the liquid-air interface, the progression of pellicle formation generally includes adhesion, conversion, growth, maturity, and detachment. These events are synchronized with the activity of bacteria in the liquid phase, which itself progresses through a lag phase and then increases in density before the surface pellicle becomes robust and the bacterial density in the liquid phase drops, as shown in Figure 2. We observe that the population of cells in the liquid phase also progress through a defined sequence of events during wild-type pellicle formation, as shown in Figure 2. We break the steps in the liquid down into 7 stages, characterized by the visual behavior of the bacteria.

1. The bacteria are inoculated in the liquid media at time *t* = 0, after which the cuvette is filled with small mobile bright spots. These likely correspond to Kobayashi's reported observation of individual cells or cell chains and clusters (Kobayashi, 2007). At 3 ± 2 h, clusters of cells begin to float on the surface of the liquid, nucleating the surface pellicle, as seen in Figure S1. The bacterial density in the liquid phase increases, simultaneous with more clusters of cells appearing on the surface of the liquid as the pellicle enters into conversion or irreversible attachment.
2. At *t* = 14 ± 3 h, streaks of high bacterial density (local OD ~ 0.12) appear throughout the liquid, typically arranged as one or two convective rolls filling the entire cuvette, as shown in **Figure 4a**. The streaks are approximately 400 um thick, and move at speeds of ~40 um/s. Additional fluorescent studies indicate that the majority of bacteria within the collective streaks express the *hag* gene, as shown in Figure S2, suggesting that the streaks are related to motility and are perhaps bioconvection (Hillesdon et al., 1995). This collective behavior has previously been reported as a haze (Kobayashi, 2007) or “vortex” (Steinberg et al., 2018) at the bottom of the liquid under the developing pellicle. At *t* ~ 17 h, the curvature of the air-liquid meniscus significantly decreases at the air-liquid-cuvette interface, indicating an abrupt decrease in air-liquid surface tension, likely associated with the production of surfactin molecules. This is consistent with previous observations reporting that *B. subtilis* uses surfactin molecules to enhance spreading of colonies by lowering the surface tension of the surrounding fluid (Angelini et al., 2009; López et al., 2009). The interface becomes progressively denser with chains of cells, forming a net of densely packed chains with motile bacteria in between, as shown in Figure S3. The chains eventually unify into a monolayer, which then thicken and become opaque, entering the growth stage of pellicle development. Around *t* ~ 25 h, the pellicle climbs the walls of the cuvette at 1.3 mm/h, likely aided by surfactin production, consistent with previous observation which attributed this behavior to surfactin production (Angelini et al., 2009; López et al., 2009).
3. The collective streaks stop, replaced by a horizontal streak of bacteria 1 ± .2 mm thick and spanning the entire width of the cuvette. At *t* = 27 ± 3 h, this streak rises toward the liquid surface, where there is now a thick pellicle. The horizontal streak rises until it reaches the pellicle, upon which it becomes uneven and wavy.
4. Sections of the stage 3 horizontal streak gradually break into top plumes, collapsing as heavy fast columns tearing away from the surface, as shown in the Supplementary Video. Eventually the horizontal streak completely disappears while the top plumes continue. These plumes are ~0.6 ± 0.2 mm thick with a dense rounded tip, and correspond to the optical density (OD) in the liquid dropping from localized streaks of *OD* = 0.12 to *OD* = 0.01 everywhere.
5. The top plumes stop, and the bacterial density within the cuvette remains low and the pellicle decelerates its climbing on the wall to 0.3 mm/h, shown by the kymograph in **Figure 5a**. Simultaneously, thin filamentous clusters of cells protrude downwards from underneath the pellicle, dangling into the liquid, as shown in **Figure 4g**.
6. At *t* = 44 ± .5 h, the pellicle disperses when plumes of matrix-associated bacteria (Figure S4) are released from underneath the pellicle on regular horizontal spatial intervals of 0.4 ± 0.2 mm. These late plumes are different in appearance from the top plumes of stage 2, low in bacterial density (OD barely above 0.01) as they settle gently into the bulk at rates ~5 mm/h, drifting side-to-side and slowly toward the bottom. For reference, a single bacterial cell falling under gravity would sink at ~ 0.8 mm/h, and an aggregate of 100 cells would fall at ~ 88 mm/h. Simultaneously, the pellicle no longer climbs the wall of the cuvette.
7. The late plumes from stage 6 continue, but at *t* = 49 ± 12 h, the pellicle matures and roughens to form wrinkles while retracting their stage 5 filaments, as can be seen in the Supplementary Video.

This completes the lifecycle of the pellicle through adhesion, conversion, growth, maturity, and finally dispersal. Synchronously, the bacteria in the liquid underneath progresses through a series of dynamics events while increasing in density, before being taken over by the mature pellicle above it.

### 3.2. Swimming and Chemotaxis and EPS Mutants Have an Altered Developmental Sequence

**Figure 3:**
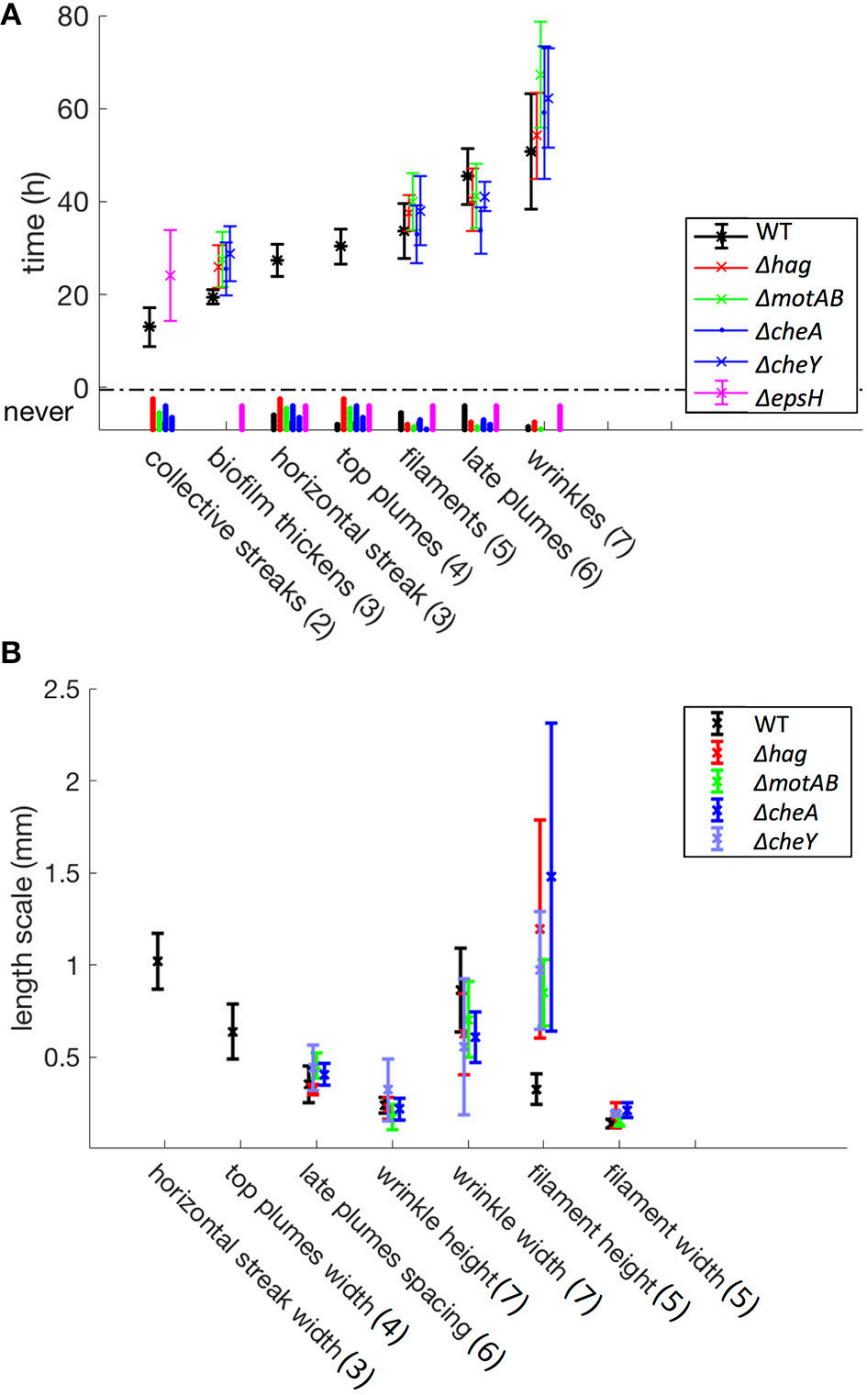
Time **(A)** and length **(B)** scales associated with the developmental events. These statistics include a total of 94 samples (37 WT, 15 Δ*hag*, 11 Δ*motAB*, 12 Δ*cheA*, 7 Δ*cheY*, and 12 Δ*epsH* mutants). Error bars represent standard deviation, and under the y-axis label “never” are histograms for mutant samples that never exhibit these developmental events. In parentheses are the corresponding stage numbers from Figure 2. The swimming and chemotaxis mutants form a pellicle later in time as compared to the wild-type, and do not exhibit collective streaks, horizontal streaks, or top plumes. However, the swimming and chemotaxis mutants have much longer filaments. The Δ*epsH* mutants exhibit coherent clouds, but no other subsequent events in the liquid bulk.

Many of the events in the liquid bulk appear dynamic and likely related to the bacteria's ability to maneuver and sense its environment. To test this, we compare the developmental cycles of wild-type pellicles to those of mutants defective in motility and chemotaxis. *B. subtilis* uses its flagella to swim and swarm, its flagella consisting of a filament of subunits of the flagellin protein, encoded by the gene *hag* (LaVallie and Stahl, 1989), and a motor subunit that enables the flagellar hook and filament rotation, formed by the proteins MotA and MotB (Mirel et al., 1992). The mode of swimming is influenced by the chemotaxis machinery, governed by the two component system CheA-CheY (Mukherjee and Kearns, 2014). We tested both motility mutants (Δ*hag*, Δ*motAB*) as well as chemotaxis mutants (Δ*cheA*, Δ*cheY*). As previously reported (Kobayashi, 2007; Hölscher et al., 2015), these strains all eventually form a pellicle that is very similar in appearance to the wild-type, although on a delayed time-scale, shown in Figure 3.

**Figure 4:**
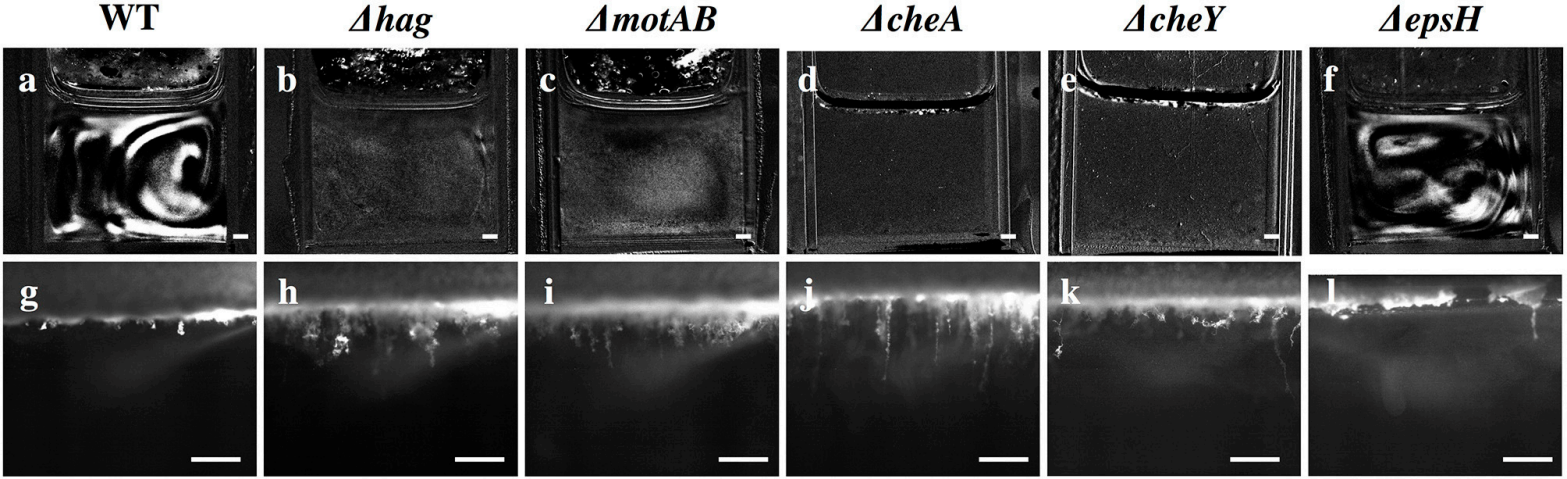
Snapshots of stage 2 collective streaks and stage 5 filaments in the liquid bulk for the wild-type and various mutants. Row 1 **(a–f)**: Collective streaks, visualized by subtracting the previous frame from the current frame. Only wild-type and Δ*epsH* have collective streaks. Scale bar is 1 mm. Row 2 **(g–l)**: Wild-type filaments are shorter than filaments for Δ*hag*, Δ*motAB*, Δ*cheA*, and Δ*cheY*. Δ*epsH* continuously tries to form a pellicle, and manages some clusters, but those clusters constantly fall and a pellicle is never properly formed. Scale bar is 1 mm.

**Figure 5:**
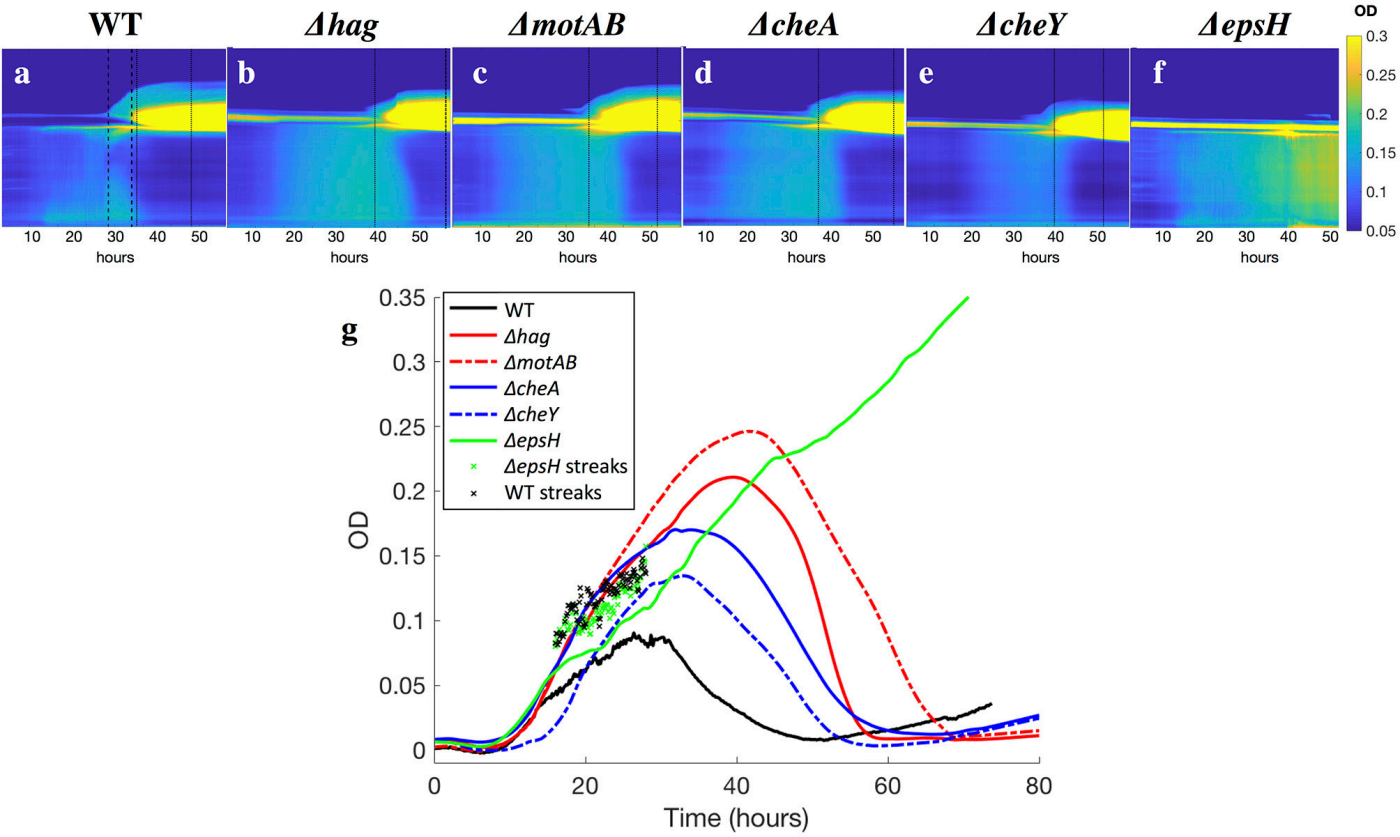
**(a–f)** Kymographs of the OD of the liquid underneath developing pellicles, for all strains. Time is plotted on the x-axis, and the y-axis is the horizontally-averaged density of vertical position in the cuvette. The bright yellow section is the surface pellicle climbing the wall. Dotted lines indicate when top plumes start and end (only in the wild-type), and dashed lines indicate when late plumes begin and end. Top plumes correlate with a drop in the bacterial density in the liquid of the wild-type, while late plumes correlate with a drop in the bacterial density of the Δ*hag*, Δ*motAB*, Δ*cheA*, and Δ*cheY* mutants. In Δ*epsH*, the bulk is never quieted, and additionally plumes do not appear. Late plumes also correspond to the pellicle no longer climbing the wall in the wild-type. **(g)** Average ODs in the liquid for all strains. Across all strains, the bacterial density rises and then drops, with the exception of the Δ*epsH* mutant, who does not form a pellicle and whose density also does not drop. Wild-type bacteria density is suppressed fastest at approximately 30 h when the average cuvette OD reaches ~ 0.08, followed by chemotaxis mutants at 35 h (OD ~ 0.15) and motility mutants at 40 h (OD ~ 0.22). In ‘x’s are the local OD's within the collective streaks of the WT and Δ*epsH* strains, which are higher locally than in the rest of the cuvette.

Despite the eventual formation of similar-looking pellicles, the progression of the developmental events is not the same for the wild-type and the mutants. Similar to the wild-type, the motility and chemotaxis mutants display early chains and clusters in the liquid bulk (stage 1). The bacteria continue to proliferate in the cuvette and increase in density while colonizing the surface, but the mutants do not exhibit the collective streaks seen in the wild-type, suggesting that motility and chemotaxis are essential to the collective streaks. Rather, the bacterial density is spatially uniform throughout the cuvette, shown in Figures 4b–e, rising until approximately OD 0.15 at *t* ~ 35 h for the chemotaxis mutants and OD 0.22 at *t* ~ 40 h for the swimming mutants. Similar to the wild-type pellicle, the meniscus increases and the pellicle unifies and thickens on the surface, shown in Figure 5, after which the pellicle begins to climb the wall.

Once a thick pellicle has formed on the surface, the OD in the liquid drops to 0.01. However, this step in the mutants is not associated with the stage 3 horizontal streak or stage 4 top plumes of the wild-type, as shown in Figure 3. Instead, this drop is associated with the shedding of thinner plumes from the bottom of the pellicle, quite similar in appearance to the stage 6 late plumes at *t* ~ 44 h in the wild-type pellicle. As compared to the wild-type, the time delay to forming a mature pellicle and suppressing the bacterial density in the liquid bulk is Δ*t* = 5 h for the chemotaxis mutants and Δ*t* = 10 h for the motility mutants, shown in Figure 3. This suggests that the streaks of high bacterial density in the wild-type facilitate faster transport of bacteria to the surface and therefore surface colonization, but is not critical for pellicle formation. Additionally, the smaller time delay of the chemotaxis mutants compared to the motility mutants suggests that the ability of the bacteria to swim, despite its lack of chemotaxis, still confers it an advantage to pellicle formation. Similarly, Hölscher et al. observed that an oxygen-sensing mutant outcompeted the *hag* mutant, again confirming that immobility is more harmful than lack of environmental sensing (Hölscher et al., 2015).

Is there a function for the stage 2 collective streaks of the wild-type? For comparison, the mutants do not exhibit collective streaks, but form a mature biofilm later than the wild-type. Notably, the chemotaxis mutants form a full pellicle and exit stage 2 when they achieve a uniform OD of 0.15 throughout the entire cuvette. The wild-type, however, achieves OD 0.12 locally within the collective streaks, while maintaining an OD of 0.08 throughout the rest of the liquid. Since it is faster for the bacteria to aggregate in local patches of high density than it is for them to reproduce to that same density everywhere, we speculate that the collective streaks of the wild-type create local pockets of high bacterial density in order to form a surface pellicle faster. The concept of different behaviors induced above and below a critical density is well-known as quorum sensing (Miller and Bassler, 2001), and has been numerically shown to affect pellicle formation and development (Ardré et al., 2015).

Finally, as in the mature wild-type pellicle, the mutant pellicles also emit filaments into the liquid and wrinkle (stages 5 and 7), but interestingly the mutant filaments are on average 3 times longer (~1.5 mm versus 0.5 mm) than those of the wild-type, as shown in Figures 3, 4g–k. The cause for the filaments being significantly longer is unclear and requires further investigation. Hence, the motility and chemotaxis mutants follow the wild-type progression of events on the interface for all stages, but deviate in events in the liquid bulk from stage 2 onwards.

Without the EPS holding it together, it is known that pellicles cannot form (Branda et al., 2005). However, it is worth examining more systematically which stages of the development are affected and which are not, as related to the EPS. The EPS is composed of expolysaccharides synthesized by the products of the *eps*A-O operons (Vlamakis et al., 2013). We therefore observed the pellicle formation process of a mutant defective in EPS production (Δ*epsH*). The Δ*epsH* mutant exhibits the same first two stages of pellicle formation, progressing from cells to chains to streaks of high bacterial density dynamically traveling around the liquid phase, shown in Figure 4f. However, while the collective streaks facilitate thickening of the wild-type pellicle, the Δ*epsH* mutants cannot form a pellicle. Chunks of pellicle still appear at the liquid surface but are never able to form one cohesive pellicle as the chunks keep forming at the surface and sinking, shown in Figure 4l. Without a robust pellicle above it, the bacteria in the liquid bulk continues as if it were a standing culture, continuing to increase in density beyond that of the other strains, as shown in Figure 5f. In all of the other mutants, a thick surface pellicle coincides with the bacterial density in the liquid bulk dropping. However, without a mature pellicle, the bacterial density in the liquid bulk continues increasing, indefinitely stuck in stage 2 of pellicle growth.

## 4. Conclusions

The general progression of pellicle formation includes bacterial adhesion, conversion, growth, maturity, and detachment to the air-liquid interface, which are synchronized with the bacteria in the liquid progressing through lag phase (chaining and clustering) and then increasing in density (collective streaks and horizontal streak and top plumes) before being taken over by the mature pellicle, after which the pellicle disperses (late plumes). These can be broken up into 7 stages, as shown in Figure 2.

With the use of mutants we have identified motility and chemotaxis as essential to the stage 2 collective streaks of high bacterial density in the liquid. These streaks do not form in the motility and chemotaxis mutants, which also take longer to form a pellicle. Of note, the chemotaxis mutants form a robust pellicle when the liquid OD is 0.15 across the entire cuvette. The wild-type, however, forms a robust pellicle when the local OD within the streaks is 0.12 and the OD everywhere else is 0.08. This leads us to speculate that the collective streaks may be a mechanism for the bacteria to create local pockets of critical bacterial density, as compared to the slower method of achieving the critical density uniformly everywhere via reproduction alone. This may enable the wild-type to achieve faster surface colonization, and therefore pellicle formation. The swimming mutants are delayed even further in robust pellicle formation (at OD 0.22), suggesting that the ability of the bacteria to swim is beneficial despite its chemotaxis abilities.

Our observations also suggest that once a robust surface pellicle is formed, the bacterial density drops in the liquid bulk. This corresponds with the appearance of collectively falling bacteria underneath the pellicle: in the wild-type these are the stage 4 top plumes. In the chemotaxis/motility mutants these are thinner plumes, similar in appearance to the stage 6 late plumes of the wild-type. When a robust surface pellicle does not form, as in the EPS mutant, the pellicle cannot progress beyond stage 2, and the bacterial density in the liquid continues increasing.

The stage 4 top plumes of the wild-type resemble bioconvection, or a collective phenomenon that occur when oxytactic bacteria (denser than water) swim to the liquid surface, and surface accumulation produces bacteria-rich plumes that fall back down away from the surface (Hillesdon et al., 1995; Jánosi et al., 1998; Metcalfe and Pedley, 2001). Given that *B. subtilis* are aerobic bacteria and denser than water, and also that swimming mutants are unable to generate top plumes, we speculate that the top plumes result from bioconvection (Hillesdon et al., 1995; Metcalfe and Pedley, 2001).

The stage 6 late plumes of the wild-type are likely matrix-bound clusters of bacteria falling from the pellicle under gravity. Since this pellicle shedding coincides with when the pellicle stops climbing the wall, the late plumes may be the pellicle entering death and undergoing pellicle dispersal. The thin plumes of the chemotaxis and motility mutants, related to the drop in bacterial density in the liquid bulk, are similar in appearance to the stage 6 late plumes of the wild-type and are probably also shedding of the pellicle.

We have observed a reproducible sequence of events that occurs with the bacteria underneath a developing liquid-air *B. subtilis* pellicle. This sequence of events is dynamic, progressing between different behaviors and patterns as the bacteria reproduces in the liquid bulk, colonizes the surface by 25 h, forms a mature surface pellicle while dropping in density within the liquid bulk by 30 h, and the pellicle disassembles by 50 h. With the use of mutants we have identified the relevant genes for specific developmental events to occur. This observation of the progression of events brings to light the complex collective dynamics occurring within pellicles as they form and mature. However, a further understanding of the biological mechanisms regulating these events and transitions is necessary to develop our understanding of biofilm formation. Understanding the mechanisms by which biofilms form, mature, and survive helps us to learn more about the collective mechanisms inherent to communities, and even multicellular organisms, as well as what enables them to become so robust.

## Author Contributions

LL and SR designed the experiments. LL and GR performed the experiments. LL analyzed the data and LL and SR wrote the manuscript.

### Conflict of Interest Statement

The authors declare that the research was conducted in the absence of any commercial or financial relationships that could be construed as a potential conflict of interest.

## References

[B1] Aguilar C. Vlamakis H. Losick R. Kolter R. (2007). Thinking about *Bacillus subtilis* as a multicellular organism. Curr. Opin. Microbiol. 10, 638–643. 10.1016/j.mib.2007.09.006 17977783 PMC2174258

[B2] Angelini T. E. Roper M. Kolter R. Weitz D. A. Brenner M. P. (2009). *Bacillus subtilis* spreads by surfing on waves of surfactant. Proc. Natl. Acad. Sci. U.S.a. 106, 18109–18113. 10.1073/pnas.0905890106 19826092 PMC2775334

[B3] Ardré M. Henry H. Douarche C. Platt M. (2015). An individual-based model for biofilm formation at liquid surfaces. Phys. Biol. 12, 066015. 10.1088/1478-3975/12/6/066015 26656539

[B4] Branda S. S. González-Pastor J. E. Ben-Yehuda S. Losick R. Kolter R. (2001). Fruiting body formation by *Bacillus subtilis*. Proc. Natl. Acad. Sci. U.S.A. 98, 11621–11626. 10.1073/pnas.191384198 11572999 PMC58779

[B5] Branda S. S. Vik S. Friedman L. Kolter R. (2005). Biofilms: the matrix revisited. Trends Microbiol. 13, 20–26. 10.1016/j.tim.2004.11.006 15639628

[B6] Dervaux J. Magniez J. C. Libchaber A. (2014). On growth and form of *Bacillus subtilis* biofilms. J. Bacteriol. 4:20130051. 10.1098/rsfs.2013.0051 25485075 PMC4213440

[B7] Flemming H.-C. Wingender J. (2010). The biofilm matrix. Nat. Rev. Microbiol. 8,623–633. 10.1038/nrmicro2415 20676145

[B8] Ghannoum M. (2004). Microbial Biofilms. Washington, DC: ASM Press.

[B9] Hillesdon A. J. Pedley T. J. Kessler J. O. (1995). The development of concentration gradients in a suspension of chemotactic bacteria. Bull. Math. Biol. 57, 299–344. 10.1007/BF02460620 7703922

[B10] Hölscher T. Bartels B. Lin Y.-C. Gallegos-Monterrosa R. Price-Whelan A. Kolter R.. (2015). Motility, chemotaxis and aerotaxis contribute to competitiveness during bacterial pellicle biofilm development. J. Mol. Biol. 427, 3695–3708. 10.1016/j.jmb.2015.06.014 26122431 PMC4804472

[B11] Jánosi I. M. Kessler J. O. Horváth V. K. (1998). Onset of bioconvection in suspensions of *Bacillus subtilis*. Phys. Rev. E 58, 4793–4800. 10.1103/PhysRevE.58.4793

[B12] Kobayashi K. (2007). *Bacillus subtilis* pellicle formation proceeds through genetically defined morphological changes. J. Bacteriol. 189, 4920–4931. 10.1128/JB.00157-07 17468240 PMC1913431

[B13] Kolter R. Greenberg E. P. (2006). The superficial life of microbes. Nature 441, 300–302. 10.1038/441300a 16710410

[B14] LaVallie E. R. Stahl M. L. (1989). Cloning of the flagellin gene from *Bacillus subtilis* and complementation studies of an in vitro-derived deletion mutation. J. Bacteriol. 171, 3085–3094. 10.1128/jb.171.6.3085-3094.1989 2498283 PMC210019

[B15] López D. Fischbach M. A. Chu F. Losick R. Kolter R. (2009). Structurally diverse natural products that cause potassium leakage trigger multicellularity in *Bacillus subtilis*. Proc. Natl. Acad. Sci. U.S.A. 106, 280–285. 10.1073/pnas.0810940106 19114652 PMC2629187

[B16] Mason J. M. Hackett R. H. Setlow P. (1988). Regulation of expression of genes coding for small, acid-soluble proteins of *Bacillus subtilis* spores: studies using lacz gene fusions. J. Bacteriol. 170, 239–244. 10.1128/jb.170.1.239-244.1988 3121585 PMC210633

[B17] McLoon A. L. Kolodkin-Gal I. Rubinstein S. M. Kolter R. Losick R. (2011). Spatial regulation of histidine kinases governing biofilm formation in *Bacillus subtilis*. J. Bacteriol. 183, 679–685. 10.1128/JB.01186-10 PMC3021239 21097618

[B18] Mendelson N. H. Bourque A. Wilkening K. Anderson K. R. Watkins J. C. (1999). Organized cell swimming motions in *Bacillus subtilis* colonies: Patterns of short-lived whirls and jets. J. Bacteriol. 181, 600–609. 9882676 10.1128/jb.181.2.600-609.1999 PMC93416

[B19] Metcalfe A. M. Pedley T. J. (2001). Falling plumes in bacterial bioconvection. J. Fluid Mech. 445, 121–149. 10.1017/S0022112001005547

[B20] Miller M. B. Bassler B. L. (2001). Quorum sensing in bacteria. Ann. Rev. Microbiol. 55, 165–199. 10.1146/annurev.micro.55.1.165 11544353

[B21] Mirel D. B. Chamberlin M. J. (1989). The *Bacillus subtilis* flagellin gene (hag) is transcribed by the sigma 28 form of rna polymerase. J. Bacteriol. 171, 3095–3101. 10.1128/jb.171.6.3095-3101.1989 2498284 PMC210020

[B22] Mirel D. B. Lustre V. M. Chamberlin M. J. (1992). An operon of *Bacillus subtilis* motility genes transcribed by the sigma d form of rna polymerase. J. Bacteriol. 174, 4197–4204. 10.1128/jb.174.13.4197-4204.1992 1624413 PMC206194

[B23] Mukherjee S. Kearns D. B. (2014). The structure and regulation of flagella in *Bacillus subtilis*. Ann. Rev. Genet. 48, 319–340. 10.1146/annurev-genet-120213-092406 25251856 PMC4869327

[B24] O'Toole G. Kaplan H. B. Kolter R. (2000). Biofilm formation as microbial development. Ann. Rev. Microbiol. 54, 49–79. 10.1146/annurev.micro.54.1.49 11018124

[B25] Romero D. Vlamakis H. Losick R. Kolter R. (2014). Functional analysis of the accessory protein tapa in *Bacillus subtilis* amyloid fiber assembly. J. Bacteriol. 196, 1505–1513. 10.1128/JB.01363-13 24488317 PMC3993358

[B26] Rubinstein S. M. Kolodkin-Gal I. McLoon A. Chai L. Kolter R. Losick R.. (2012). Osmotic pressure can regulate matrix gene expression in *Bacillus subtilis*. Mol. Microbiol. 86, 426–36. 10.1111/j.1365-2958.2012.08201.x 22882172 PMC3828655

[B27] Seminara A. Angelini T. E. Wilking J. N. Vlamakis H. Ebrahim S. Kolter R.. (2012). Osmotic spreading of *Bacillus subtilis* biofilms driven by an extracellular matrix. Proc. Natl. Acad. Sci. U.S.A. 109, 1116–1121. 10.1073/pnas.1109261108 22232655 PMC3268299

[B28] Shapiro J. A. (1998). Thinking about bacterial populations as multicellular organisms. Ann. Rev. Microbiol. 52, 81–104. 10.1146/annurev.micro.52.1.81 9891794

[B29] Srinivasan S. Vladescu I. D. Koehler S. A. Wang X. Mani M. Rubinstein S. M. (2018). Matrix production and sporulation in *Bacillus subtilis* biofilms localize to propagating wave fronts. Biophys. J. 114, 1490–1498. 10.1016/j.bpj.2018.02.002 29590605 PMC5883974

[B30] Steinberg N. Rosenberg G. Keren-Paz A. Kolodkin-Gal I. (2018). Collective vortex-like movement of *Bacillus subtilis* facilitates the generation of floating biofilms. Front. Microbiol. 9:590. 10.3389/fmicb.2018.00590 29651280 PMC5884953

[B31] Stoodley P. Sauer K. Davies D. G. Costerton J. W. (2002). Biofilms as complex differentiated communities. Ann. Rev. Microbiol. 56, 187–209. 10.1146/annurev.micro.56.012302.160705 12142477

[B32] Sutherland I. (2001). Biofilm exopolysaccharides: a strong and sticky framework. Microbiology 147, 3–9. 10.1099/00221287-147-1-3 11160795

[B33] Vlamakis H. Chai Y. Beauregard P. Losick R. Kolter R. (2013). Sticking together: building a biofilm the *Bacillus subtilis* way. Nat. Rev. Microbiol. 11, 157–168. 10.1038/nrmicro2960 23353768 PMC3936787

[B34] Wang X. Koehler S. A. Wilking J. N. Sinha N. N. Cabeen M. T. Srinivasan S.. (2016). Probing phenotypic growth in expanding *Bacillus subtilis* biofilms. Appl. Microbiol. Biotechnol. 100, 4607–4615. 10.1007/s00253-016-7461-4 27003268

