# Supplementary material for: A Sequence of Developmental Events Occurs Underneath Growing Bacillus subtilis Pellicles
Source: Front Microbiol. 2019 Apr 26;10:842. doi: 10.3389/fmicb.2019.00842 (PMC6499031; doi:10.3389/fmicb.2019.00842)
Supplement: Supplementary file 1 [file Data_Sheet_1.pdf]

Supplementary Material

1 OD MEASUREMENT TECHNIQUE

OD measurements of the bacteria in the liquid underneath the pellicles were conducted. The setup was built into the time-lapsing multi-cuvette turntable setup. A green LED was passed through an aperture and a spherical lens (focal length $\approx$3 inches) and positioned $\sim$ 17 inches from the back of the cuvette for a relatively collimated light source by the time it reached the cuvette. The light passing through the cuvette was then collected as an image by the PCO edge 4.2 SCMOS camera with a Canon Macro lens. These images were then analyzed for spatially resolved OD measurements throughout the liquid underneath the growing pellicle.

Image pixel intensities were calibrated to OD values by growing a very dense suspension of bacteria (OD 1.37) and measuring its OD in a commercial OD meter (Biowave CO8000 Cell Density Meter) measuring at 600 nm. The suspension was then imaged in our setup, and the pixel intensity values recorded. The suspension was then progressively diluted to lower bacterial densities, and at each dilution we measured both its OD using a commercial OD meter as well as its pixel intensity values in our setup. These measurements were then used to provide a calibration curve between pixel intensity value and OD.

2 VIDEO OF DEVELOPMENTAL SEQUENCE EVENTS

Included (WTsequenceLabeled.avi). Time-lapse through the side of the cuvette. This video was obtained using a back-lit setup, hence darker regions correspond to higher cell density. The video progresses through the 7 steps discussed in the paper.

3 FIGURES

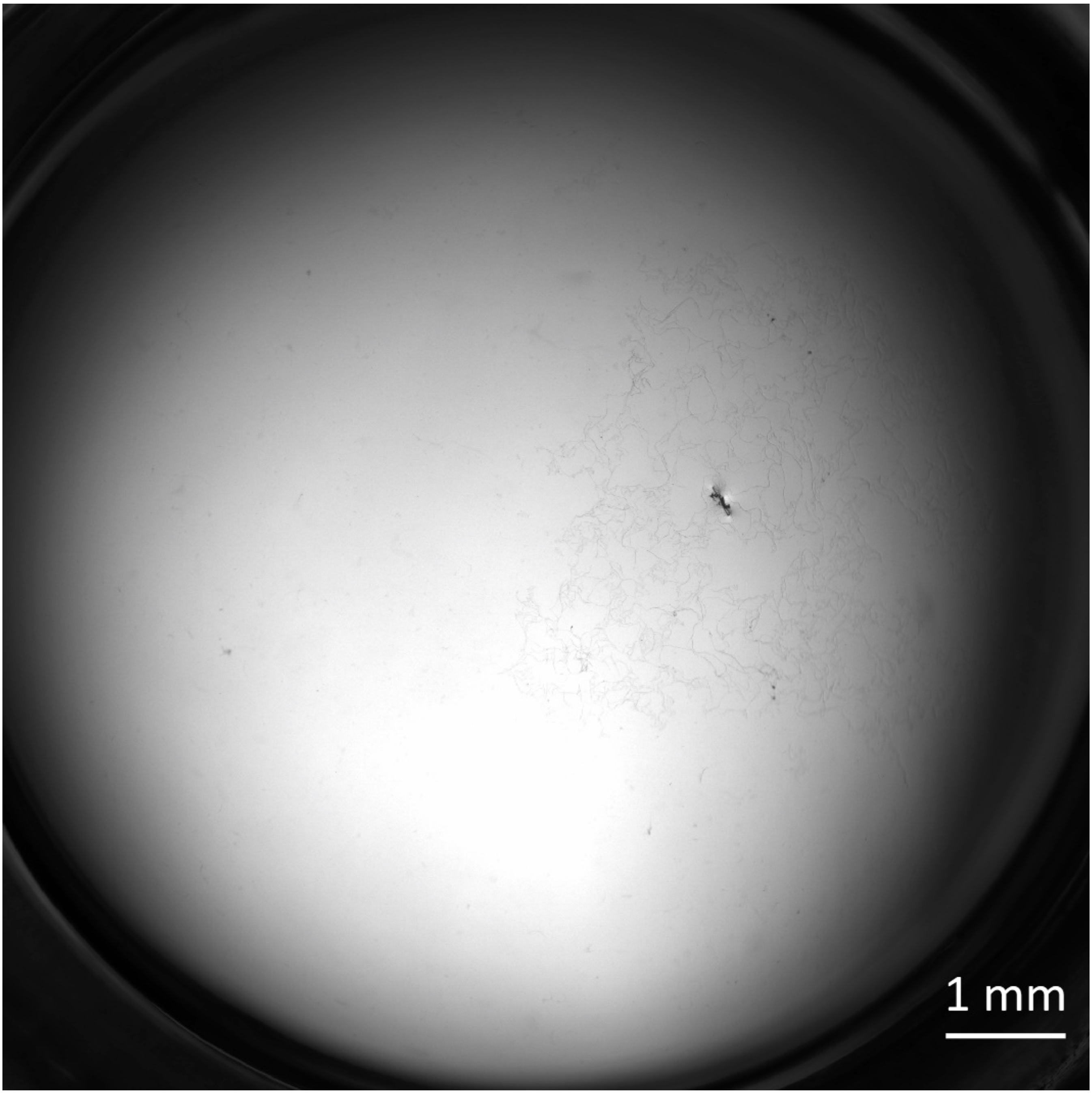

Figure S1. Chunks/chains of cells float on the surface to nucleate the surface pellicle.

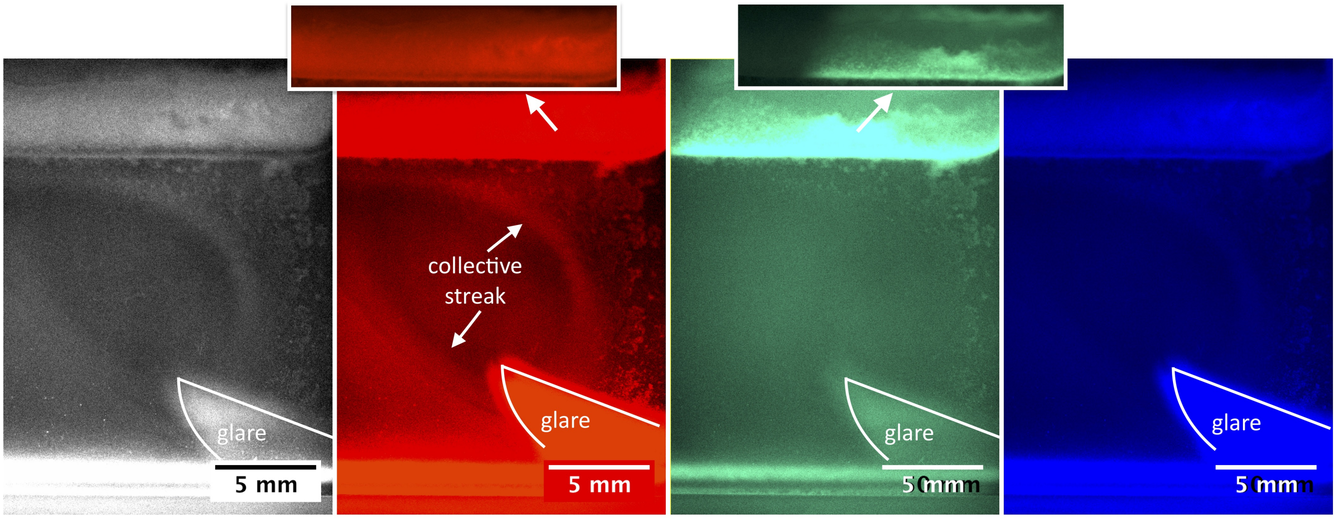

(a) Brightfield (b) Motility reporter (c) EPS Matrix reporter (d) Sporulating reporter

Figure S2. Side view of the cuvette. The stage 2 collective streaks of the triple-reporter under fluorescence. The collective clouds fluoresce in the motility (*hag*) reporter channel. The fluorescent glare in the lower right corner is labeled and is not part of the collective streaks. For (b) and (c), insets show the pellicle under altered brightness thresholds in order to better view the pellicle.

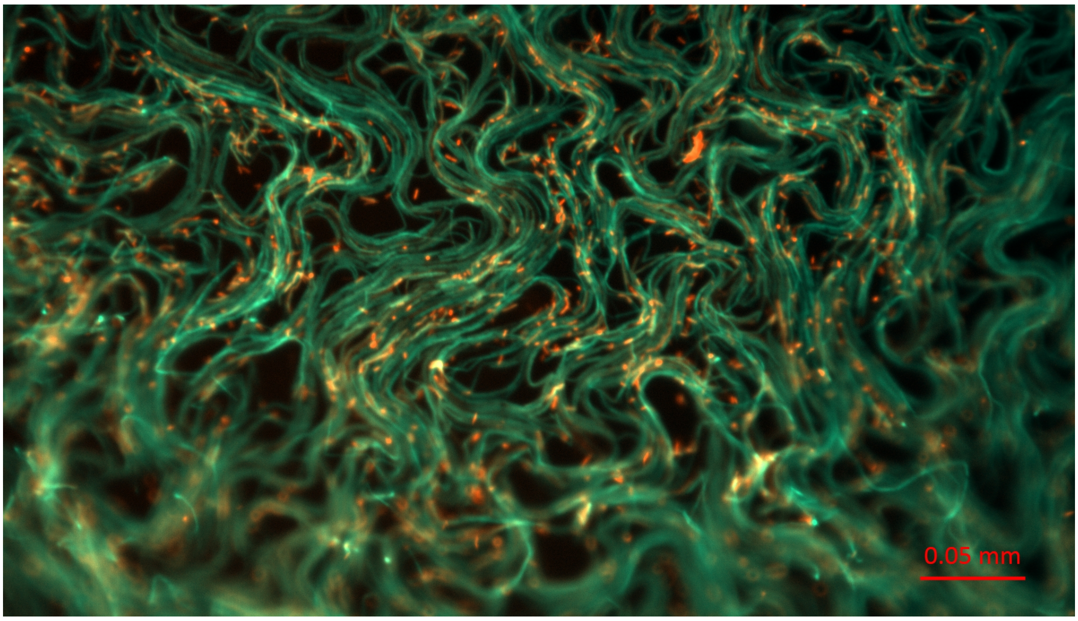

Figure S3. The chained (*tapA-sipW-tasA*) bacteria cells in green, at the air-liquid interface when the pellicle is still becoming a monolayer. In red are the motile (*hag*) cells swimming around and through the chains. The bacterial strain is the triple-reporter strain mentioned earlier. This fluoresence image was acquired on a Zeiss Axiozoom V16 microscope with a PlanNeoFluar 2.3x lens, using a Hamamatsu SCMOS imaging sensor.

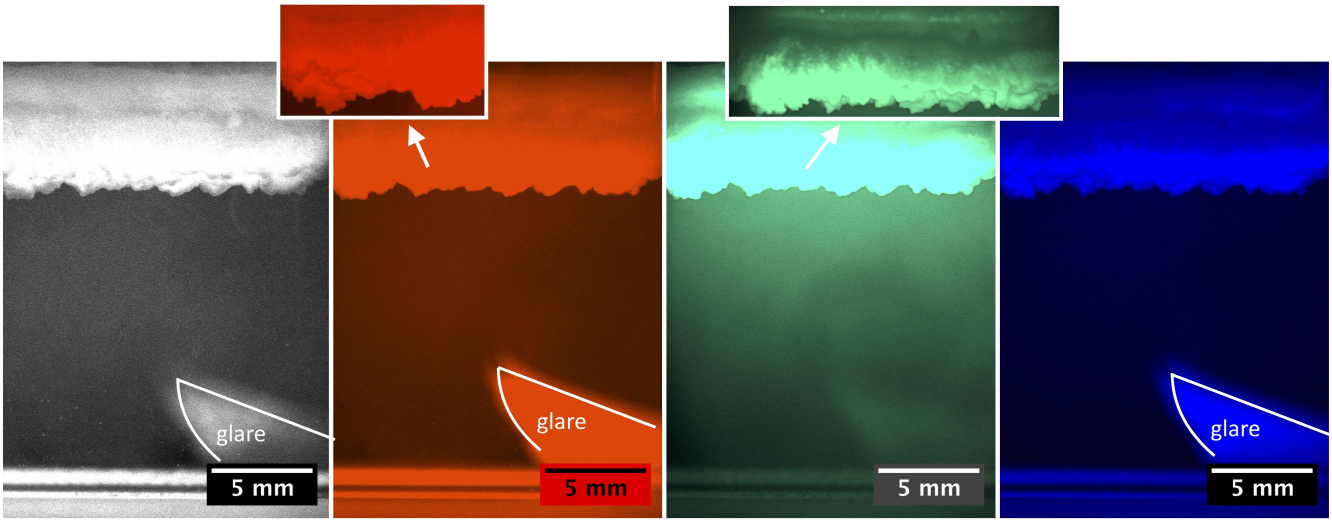

(a) Brightfield (b) Motility reporter (c) EPS Matrix reporter (d) Sporulating reporter

Figure S4. Side view of the cuvette. The stage 6 late plumes of the triple-reporter under fluorescence. The bacteria in the liquid is brighter at the top in the EPS matrix (*tapA-sipW-tasA*) reporter channel. For (b) and (c), insets show the pellicle under altered brightness thresholds in order to better view the pellicle.

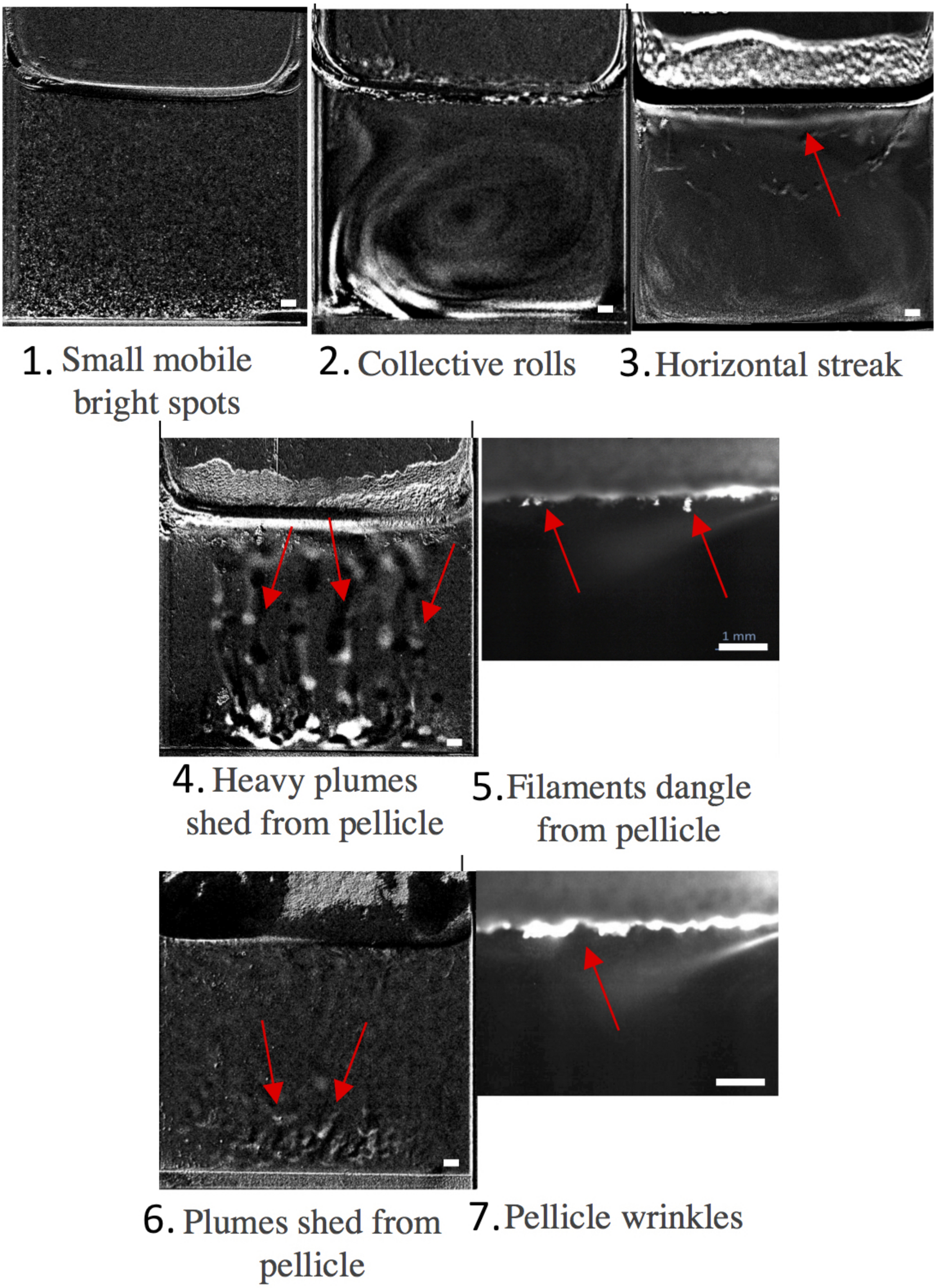

Figure S5. Full photographic views of the stages from Fig 2 of the main text.
